# The Bristol Breastfeeding Assessment Tool (BBAT): Α systematic review of the psychometric properties of the translated versions

**DOI:** 10.18332/ejm/201343

**Published:** 2025-02-24

**Authors:** Pinelopi Varela, Christina Nanou, Maria Bouroutzoglou, Giannoula Kyrkou, Anna Deltsidou

**Affiliations:** 1 General Hospital of Athens ‘Alexandra’, Department of Midwifery, University of West Attica, Athens, Greece; 2 Department of Midwifery, University of West Attica, Athens, Greece; 3 Department of Midwifery, International Hellenic University, Thessaloniki, Greece

**Keywords:** Bristol Breastfeeding Assessment Tool, breastfeeding, breast milk, infants, neonate, systematic review

## Abstract

**INTRODUCTION:**

The Bristol Breastfeeding Assessment Tool (BBAT) has gained the interest of healthcare professionals involved in breastfeeding. The aim of this systematic review is to assess the psychometrics of the translated versions of the BBAT.

**METHODS:**

The databases PubMed, Scopus, Science Direct, and DOAJ were used to conduct a search for articles published between 2015 and 2023. The PRISMA guidelines were followed for the conduct and reporting of the review, and the COSMIN checklist was utilized to evaluate the psychometrics of the studies that were retrieved.

**RESULTS:**

Of the 117 records initially identified, four studies were included. The analysis of these studies revealed that the scale is available in at least four different languages. The methodological quality of the structural validity reported by three studies was satisfactory. Only two studies provided information on test-retest reliability, while the majority of the studies demonstrated very good quality in terms of internal consistency. All studies examined the construct validity of the BBAT, and the methodological quality produced different outcomes.

**CONCLUSIONS:**

The methodological quality of the psychometrics of the translated versions of the BBAT provided mixed results. The continuation of the validation of the scale in more languages is recommended.

## INTRODUCTION

The normative standards for the optimal nutrition and feeding of newborns are breastfeeding (BF) and human breast milk^1^. BF is the biological norm and the best source of nutrition for newborns since it is free, hygienic, and ideally matched to their needs^2,3^. Exclusive BF for the first six months of life is recommended by the World Health Organization (WHO) and the United Nations Children’s Fund (UNICEF)^2,3^.

The existence of these recommendations is due to evidence indicating that BF offers multiple benefits^4,5^. One of these is the reduced risk of several infectious diseases^6^ and it is anticipated that BF will result in a 13% decrease in child mortality from preventable diseases, particularly for children under five years old^5^. Also, infants, that are breastfed, experience improved brain development in addition to improved growth^7^. Additionally, research indicates that infants who are not breastfed have a three- to four-fold increased chance of dying compared to those who have exclusive BF practices^2,8,9^.

However, global breastfeeding rates have not yet attained acceptable levels, despite the fact that the benefits of BF have been well established and multiple programs supporting it exist^10^. According to the WHO, between 2007 and 2014, 36% of newborns worldwide, between the ages of 0 and 6 months, were exclusively breastfed^11^. In the United States, 81% of newborns in 2013 and 83.1% of newborns in 2020 were breastfed when they were born, but only 44.3% of them in 2013 and 45.3% of them in 2020 were exclusively breastfed by the time they were three months old^12^. Worldwide, 44% of infants are exclusively breastfed; this percentage varies from 26% in North America to 57% in South Asia. However, by the year 2030, the WHO anticipates that every member nation will have at least 70% of infants breastfed for the first six months of their life^13^.

The literature reveals a number of reasons and factors that lower BF rates and maintain them there, as well as factors that contribute positively to BF rates. Studies have indicated that not initiating BF with the first child, having difficult BF experiences, and failed efforts are linked with failure to initiate BF at subsequent deliveries^14,15^. In a recent systematic review and meta-analysis, increased BF initiation and continuation were observed for non-smokers compared to smokers, for vaginal delivery compared to cesarean delivery, and for the maternal highest level of education compared to the lowest level of education. Additionally, it was found that multiparity and dyad connections (skin-to-skin or rooming-in) were positively associated with BF initiation and maintenance^16^. Other factors that may hinder exclusive BF include nipple problems with pain, a lack of BF self-efficacy, and challenges in providing for their infants^17^. The mother’s faith in her ability to breastfeed her child and the actual BF assistance provided by midwives are important and crucial components in the initial stages of BF^18,19^. Effective BF results from the positioning, latch, sucking, and milk transfer, all of which are objective indicators of successful BF^20,21^.

The routine evaluation and observation of BF components by midwifery professionals is a fundamental strategy to detect potential issues and suggest clinical or educational interventions to mothers. Healthcare professionals in midwifery settings have the possibility of using an assessment instrument for BF as a reference. The use of an instrument can facilitate regular evaluation and simplify the process of determining the exact timing and course of action as required, which could therefore positively impact BF rates. Several assessment tools are available to guide the clinical assessment of BF. These scales are intended to identify mothers who may be at risk of quitting BF and provide them with further support, when necessary, by identifying the existence of issues. Additionally, simplified information sharing across midwifery professionals is made possible by the use of these scales^22-25^.

The most current, published instrument is the Bristol Breastfeeding Assessment Tool (BBAT)^26^. The BBAT was created for healthy, full-term infants and was tested on 218 BF sessions with infants up to 10 weeks old. It is composed of four items that evaluate the four essential elements of feeding: positioning, attachment, sucking, and milk transfer. There is a note attached to each item that details the characteristics of each of these components to observe for. Every item has the following rating: poor (0 points; not meeting the requirement for the feature), moderate (1 point), and good (2 points; achievement that is successful), in order for the possible overall score to vary between zero and eight points. The internal consistency reliability of the BBAT was good (Cronbach α = 0.668), and the inter-rater reliability was high (intraclass-correlation coefficient-ICC = 0.782). Also, the BBAT showed a moderate correlation (r = 0.57) with the Breastfeeding Self-Efficacy Score (shortform) (BSES-SF)^27^. In comparison to the LATCH tool^28^ and Infant Breast-Feeding Assessment Tool (IBFAT)^29^, the BBAT was tested alongside them and was shown to be more responsive to changes over time. The developers of the tool mentioned that the components of the BBAT are relevant to a broad spectrum of infant ages and are also sensitive to the subtle changes that are critical for enhancing BF after a frenotomy^26^.

Although the BBAT is a relatively new tool for the assessment of BF, it has stimulated interest among health professionals involved in BF in clinical settings. This results in its translation, as well as its use in different countries. While it is becoming more popular, a systematic assessment of the psychometric properties of its translated versions is considered beneficial. The evaluation of the preliminary findings of the studies that validated the BBAT may offer valuable information to healthcare professionals who work in midwifery settings and deal with the BF process. The gathered research data that will result from the present review can be useful for upcoming BBAT validations. Thus, the first objective of the current systematic review was to systematically identify the translated versions of the BBAT, and the second was to evaluate the psychometric properties of the translated versions.

## METHODS

The Preferred Reporting Items for Systematic Reviews and Meta-Analyses (PRISMA) statement’s recommendations^30^ were followed in conducting the current systematic review. There is no registration information, and the review was conducted using an *a priori* protocol that outlined the process to be followed.

### Eligibility criteria

The PICOS framework, which is a model for structuring research questions, was used to apply the inclusion and exclusion criteria. The PICOS method is specified by describing the population/participants (P), the intervention (I) and comparator (C) of interest, the outcomes (O), and the study design (S). Thus, the inclusion criteria of the current study were studies in BF women that used the BBAT tool and that reported the psychometric properties of the translated versions of the BBAT. In addition, only peer-reviewed English-language published studies that were released between 2015 and 2023 with no restriction regarding the geographical location were included. The exclusion criteria were studies that did not involve BF women and that did not use the BBAT tool. Additionally, studies that used the BBAT as an outcome measure and did not report the translation process, and afterwards, the psychometric properties of the translated version of the BBAT as well as letters, commentaries, research protocols, reviews, meta-analyses, and editorials, were excluded.

### Information sources

We searched for peer-reviewed articles in PubMed, Scopus, Science Direct, and DOAJ. With the last search being conducted on 7 November 2023, the search results covered the period from 2015 (the year of publication of the original BBAT instrument) through November 2023. Reference lists of the included articles were screened for additional articles that were not yielded by the initial search.

### Search strategy

The keywords used were: ‘Bristol Breastfeeding Assessment Tool’, ‘BBAT’, ‘reliability’/‘reliab*’, and ‘validation’/‘valid*’. In each database, the following algorithm was used: [X]≡[Bristol Breastfeeding Assessment Tool OR BBAT], [X AND reliab*], [X AND reliability], [X AND valid*], and [X AND validation]. The following search filters were used in the PubMed database: article language: English and sex: female. The Scopus database was searched with the English article language filter. The search parameters for research papers, English article language, and subject areas of nursing and health professions were applied to the Science Direct database.

### Selection process

Based on eligibility and exclusion criteria, two of the authors (VP and BM) independently assessed the abstracts and titles. Following that, full texts of potentially related studies were collected for additional evaluation. The rate of inter-reviewer agreement was measured by the percentage agreement and this was 80%. Disagreements among the authors on inclusion were resolved by discussion.

### Data collection process, extraction and items

A data extraction form was created by one of the authors (VP) and assessed by three co-authors (DA, BM, and NC). Following that, data were separately extracted from the retrieved studies by two of the authors (VP and DA). Any conflict was resolved by consensus among all authors.

For each study, data extraction comprised: information about each article (authors; year; country; translation language; research setting; sample characteristics; inclusion and exclusion criteria; sample size; the questionnaire’s completion period of time), translation process, and psychometric properties.

### Risk of bias in individual studies

The included studies were assessed for their methodological quality according to the guidelines by COnsensus-based Standards for the selection of health status Measurement INstruments (COSMIN) methodology for systematic reviews of Patient-Reported Outcome Measures (PROMs)^31^. This checklist consists of ten boxes /subscales, that evaluate the methodological quality of the psychometric properties. These ten subscales consist of: 1) PROM development; 2) Content validity; 3) Structural validity; 4) Internal consistency; 5) Cross-cultural validity\Measurement invariance; 6) Reliability; 7) Measurement error; 8) Criterion validity; 9) Hypotheses testing for construct validity; and 10) Responsiveness. Every box comprises typical questions required to assess the quality of a study on that specific measurement feature. Methodological quality is rated as either very good, adequate, doubtful, or of inadequate quality. The study’s overall quality is determined by taking any standard with the lowest ranking in the box (i.e. ‘the worst score counts’ principle). Each study’s findings on a measurement property should then be evaluated in light of the updated criteria for good measurement properties. Each result is rated as either sufficient (+), insufficient (–), or indeterminate (?)^31^.

In the present study, each article’s methodological quality was evaluated based on four properties: structural validity, internal consistency, reliability, and hypotheses testing for construct validity. Using the rating principles mentioned above, we rated each measurement property and the result of each study on a measurement property.

Construct validity methodological quality assessment was carried out based on the number of generic hypotheses provided in the checklist manual (i.e. Hypothesis 1: Correlations with instruments measuring similar constructs should be ≥0.50; Hypothesis 2: Correlations with instruments measuring related, but dissimilar constructs should be lower, i.e. 0.30–0.50; and Hypothesis 3: Correlations with instruments measuring unrelated constructs should be <0.30)^31^. Hypotheses 1 and 2 were used in the present review. More precisely, the review team, after the selection of the included studies, defined the following hypotheses: Hypothesis 1 pertains to the LATCH assessment tool^28^, while Hypothesis 2 concerns the short-form Breastfeeding Self-Efficacy Score (BSES-SF)^27^.

The method of tabulation was used for the presentation of characteristics and results of methodological quality from the included studies. All the data obtained were tabulated based on the data extraction form created during the initial stages of the study and based on the four pre-decided measurement properties.

## RESULTS

### Study selection

**Figure 1:**
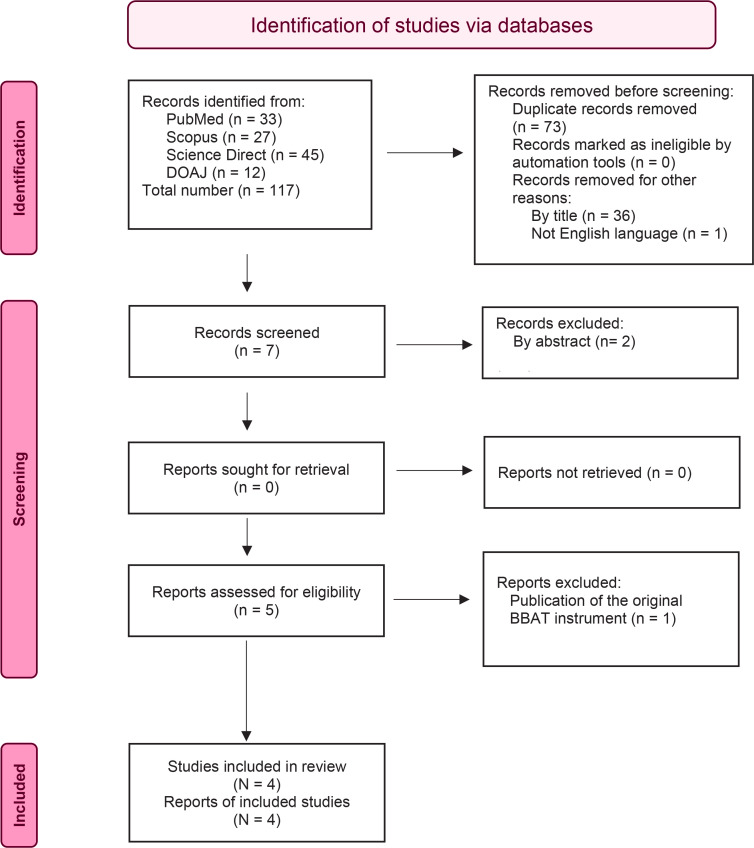
PRISMA flow diagram showing selection of the included studies

The systematic search resulted in 117 studies. Following the removal of duplicates, 44 studies remained to be screened. After exclusions of the title or abstract of the article and articles not in English, five full-text studies remained to be assessed. One study from these was excluded because it was the publication of the original BBAT instrument. The above studies’ exclusions resulted in four studies for inclusion in the present systematic review. Figure 1 shows the PRISMA flow diagram of the selection process.

### Study characteristics

The versions that are included concern four countries (Turkey, Spain, Thailand, Switzerland)^32-35^. After analyzing these four studies, it was found that the BBAT is available in four other languages (Turkish, Spanish, Thai, German)^32-35^ with the exception of English, which is the scale’s original language. Every study was conducted in a clinical environment and the postpartum women who participated were nulliparous or multiparous. The sample size of the studies ranged from 44 to 302 participants. The time frame for completing the BBAT varied from 12 hours to 24 weeks following childbirth. Table 1 provides a summary of the features of the included studies. Three studies examined the structural validity, and the results showed that the quality was either sufficient or insufficient. The majority of the studies presented very good quality in terms of their internal consistency, and only two studies reported information on test-retest reliability. The BBAT’s construct validity was examined in all of the studies, and the methodological quality yielded varying results. The methodological quality and ratings of the psychometric properties of the included studies are summarized in Table 2.

**Table 1:** Studies characteristics included in the systematic review

*Authors* *Year* *Country* *Language*	*Setting*	*Sample characteristics*	*Inclusion and Exclusion criteria*	*Sample size*	*Questionnaire’s completion period of time*
Dolgun et al.^32^ 2018 Turkey Turkish	One university hospital	Maternal mean age: 31.55 years. 52% of the participants were graduates of high school or higher education, and 55.9% had a breastfeeding education. The majority (75.6%) had a cesarean section. Infant’s mean age in weeks: 6.52. The majority (49.6%) of infants were being exclusively fed breast milk.	*Inclusion criteria:* consent to participate in the study; had fluency in Turkish; had childbirth at the 37th gestational week or later; children were between 0–6 months old; and continued to breastfeed. *Exclusion criteria:* any congenital anomalies in the infant and any chronic disease in either the mother or their neonates.	n=127 Mothers with their neonates	From 0.5 to 24 weeks
Balaguer-Martínez et al.^33^ 2018 Spain Spanish	Three primary care centers	Maternal mean age: 31.7 years. The majority (54.8%) had a secondary educational level, and 40.3% had previous breastfeeding experience. The median gestational age was 40 weeks, and the majority (74.2%) had a spontaneous vaginal birth.	*Inclusion criteria:* maternal age ≥18 years; term delivery (≥37 weeks of gestation); and exclusive breastfeeding at the time of the initial visit to the care center. *Exclusion criteria:* maternal language barrier; twin birth; and presence of congenital anomalies.	n=62 Mothers with their neonates	Within 15 days after childbirth; 1st completion: at the 1st visit of the newborn (mean days of life: 8.3) 2nd completion: after 7–10 days.
Singhala et al.^34^ 2018 Thailand Thai	Two tertiary general hospitals	Maternal mean age: 26.2 years. The majority (56.6%) had a secondary education level, were multiparous (62.6%), had a vaginal delivery (84.4%), and had no perceived education about breastfeeding (72.6%).	*Inclusion criteria:* maternal age ≥18 years; single pregnancies; uncomplicated vaginal birth; gravidity ≥1; parity ≥0; capable of giving consent; married; fluency in Thai; expressed willingness to participate; gestational age at birth ≥37 weeks; infant’s weight ≥2500 g; Apgar score ≥7 at 1 minute. *Exclusion criteria:* mothers with an underlying disease or condition; birth of twins or more; postpartum complications.	n=302 Mothers with their neonates	Within 24–36 hours of birth
Pujos et al.^35^ 2018 Switzerland German	One Swiss university hospital	Maternal median age: 34 years. The majority (67%) were multiparous and had a vaginal delivery.	*Inclusion criteria:* maternal age ≥18 years; intention to breastfeed; childbirth between the 37th and 42nd gestational weeks of pregnancy; healthy term neonates with a birthweight between 2.500 and 4.500 g. *Exclusion criteria:* gestational diabetes; perinatal blood loss >1.500 mL; transferred from other hospitals; or with a hospital stay at another ward other than the maternity ward.	n=44 Mothers with their neonates	No earlier than 12 hours after birth

**Table 2:** Methodological quality and ratings* of quality of psychometric properties of studies according to COSMIN

*Authors* *Year* *Country*	*Structural validity*		*Internal consistency*	*Reliability (test-retest)*	*Hypotheses testing for construct validity*
	*Method quality*	*Method used*			
Dolgun et al.^32^ 2018 Turkey	Very good (+)	Confirmatory factor analysis (CFA)	Very good (+)	Doubtful (+)	Very good (+) (in line with hypothesis 1)
Balaguer-Martínez et al.^33^ 2018 Spain	Adequate (-)	Principal component analysis (PCA)	Very good (+)	Adequate (-)	Inadequate (-) (in line with hypothesis 1) Inadequate (-) (in line with hypothesis 2)
Singhala at al.^34^ 2018 Thailand	Very good (+)	Confirmatory factor analysis (CFA)	Very good (+)	N/M	Adequate (-) (in line with hypothesis 2)
Pujos et al.^35^ 2018 Switzerland	Inadequate (-)	N/M	Inadequate (-)	N/M	Inadequate (-) (in line with hypothesis 2)

* Each result is rated as either sufficient (+), insufficient (-), or indeterminate (?). N/M: not mentioned.

### Translation process

In all studies, the translation process adhered to the forward-backward translation scheme, i.e. from English to Turkish^32^, to Spanish^33^, to Thai^34^, and to German^35^. The study researchers^32^ and the authors of the translated version that was published^33^ carried out the forward translation, while a professional translator^32^ and a bilingual person^33^ completed the backward translation, based on the information provided by the translated versions that have been published. The Turkish version was reviewed by an expert committee composed of seven specialists who were fluent in English and Turkish. Afterwards, the needed revisions of the scale were made in accordance with the views of the experts, and then the scale was translated into the original language by a professional translator who was not a specialist in the field^32^. For the Thai version, technical, criterion, and conceptual equivalences were performed^34^.

### Test of the prefinal version and confirmation by the developer

The Spanish version of the BBAT was pretested by five professionals in order to verify the correct comprehension of the text and was also reviewed by the developer^33^. The Turkish and Thai versions were verified by the developer^32,34^.

### Structural validity

The methodological quality of the structural validity of the Turkish version was very good, with a sufficient rating. A confirmatory factor analysis (CFA) was performed, and the goodness of fit indexes and the chi-squared value showed good and acceptable fit since they were at the intended levels^32^. Principal component analysis (PCA) was used to investigate the structural validity of the Spanish version and confirmed that the scale is unidimensional. The methodological quality of the structural validity was adequate, with an insufficient rating since CFA was not performed^33^. With a sufficient rating, the Thai version’s structural validity exhibited very good methodological quality. The results of the CFA showed a perfect fit and were entirely saturated^34^. The methodological quality of the structural validity of the German version was inadequate, with an insufficient rating since no exploratory or confirmatory factor analysis was performed^35^.

### Internal consistency

The Turkish, Spanish, and Thai versions all demonstrated very good methodological quality of the internal consistency, receiving a sufficient rating^32-34^. The methodological quality of the internal consistency of the German version was inadequate, with an insufficient rating since no Cronbach’s alpha and no item-total correlations were calculated^35^.

### Test-retest reliability

The methodological quality of the reliability of the Turkish version was doubtful, and the rating of quality was sufficient. Regarding test-retest reliability, the authors did not mention whether or not the test settings were similar, whether or not the participants were stable, and the time interval was not specified^32^. In the Spanish version, the test-retest reliability was assessed using an 8.3-day interval time and a sample of 36 participants. Although the rating of quality was insufficient, the methodological quality of reliability was adequate^33^. Regarding the Thai and German versions’ test-retest reliability, no information was provided. However, interrater reliability was confirmed in the Thai version^34^ and indicated good reliability in the German version^35^.

### Construct validity

The methodological quality of the construct validity of the Turkish version was very good, with a sufficient rating in line with hypothesis 1^32^. According to hypotheses 1 and 2, the methodological quality of the construct validity of the Spanish version was inadequate, but the quality rating was sufficient^33^. With regard to hypothesis 2, the construct validity’s methodological quality in the Thai version was adequate, with an insufficient rating^34^. Based on hypothesis 2, the methodological quality of the construct validity of the German version was inadequate, with an insufficient rating, since no information on the measurement properties of the comparator instrument was provided^35^.

## DISCUSSION

The present systematic review found four studies regarding the BBAT, which concerned translation and assessing its psychometric properties in different languages. The original English version of the tool has been translated into four languages (Turkish, Spanish, Thai, and German)^32-35^.

### Translation process, test of the prefinal version and confirmation by the developer

The forward-backward translation scheme was the chosen method in all translated versions of the BBAT, which is the optimal one. During the translation process, the review of the final translation by an expert committee is a critical step. This team of experts ought to be multidisciplinary (i.e. translators, methodologists, language professionals, and health professionals) and would need to make selections in four different areas: semantic equivalence, idiomatic equivalence, experiential equivalence, and conceptual equivalence^36^. The present study observed that only one study reported information regarding the expert committee^32^. The test of the prefinal version is the next stage of the translation process, and its goal is to evaluate the meaning and cultural significance of the items and the ease of understanding^36^. The present review observed that only one study tested the prefinal version^33^. The majority of the translated versions were verified by the tool’s developer, even though not all translation phases were followed. This conclusion is drawn from the data that the article authors reported. It remains uncertain whether direct correspondence occurred between them and the scale’s developer. However, given the information we have, it can be observed that not following all or the majority of the steps of the translation process may result in an inferiorquality version of the scale. Phases that are left incomplete could compromise the quality of the translation process and increase the likelihood that the translated version will not be accurate to the original one.

### Structural validity

The process of determining the statistical evidence for a translated scale’s validity is referred to as factorial validity and the absence of CFA is a major shortcoming for the level of evidence for structural validity^37^. In the present study, it was observed that only two studies performed CFA^32,34^ and one study performed PCA^33^, and the method quality was satisfactory in all cases.

### Internal consistency and test-retest reliability

Internal consistency was the first type of reliability measure assessed in the translated versions of the BBAT. Every study, with the exception of one^35^, mentioned a Cronbach’s alpha and had a very good methodological quality of internal consistency. However, a high degree of internal consistency is not always indicated by a high Cronbach’s alpha^38^. Test-retest reliability was the second type of reliability measure assessed. This measure provides more evidence that an outcome measure is stable over time^31^. Unfortunately, half of the studies, i.e. two studies^32,33^, reported information on test-retest reliability. However, the other two studies^34,35^ reported information on interrater reliability.

### Construct validity

The review team of the present study used hypotheses 1 and 2, which are provided in the manual of the COSMIN checklist and described in the Methods section. The results revealed from the hypotheses testing for construct validity were mixed. The LATCH assessment tool^28^ and the BSES-SF scale^27^ were the instruments used by the authors of the translated versions. Using these tools is seen as reasonable and somewhat expected. The LATCH is a five-item assessment of latch, audible swallowing, type of nipple, comfort of breast/nipple, and positioning^28^. The BSES-SF measures a mother’s confidence in her ability to BF her child and can be used clinically to identify those at high risk of discontinuing BF^27^. The psychometric characteristics of the comparator instruments were not reported in all of the studies, which is likely to have had an impact on the construct validity of the translated versions of the BBAT.

### Limitations

The initial limitation of the current study is due to the fact that the literature search was limited to four databases and that the included studies were only available in English. The above facts may have led to potentially missing relevant information. Moreover, the present results may be affected due to the absence of the level of evidence based on the Grading of Recommendations Assessment, Development, and Evaluation (GRADE). On the other hand, one of the strengths of the current study is the fact that it was conducted under established guidelines (PRISMA) for conducting systematic reviews. Additionally, the COSMIN guidelines for systematic reviews of patient-reported outcome measures were used for the assessment of the methodological quality of the included studies.

## CONCLUSIONS

The BBAT has been translated into at least four languages and is of interest to healthcare professionals. The authors of the translated versions mentioned that each adopted version of BBAT is reliable and valid. However, the methodological quality of the psychometric properties of the translated versions reviewed in the present study provided mixed results. More specifically, these varied results mainly concern the test-retest reliability and the construct validity of half of the studies.

Future studies are strongly encouraged to utilize and adhere to standards for the translation and cultural adaptation process, meeting as many of these guidelines’ stages as feasible. Also, further research on the structural validity of the BBAT is necessary. Authors of upcoming studies should describe in detail not only the comparator instruments but also their psychometrics.

## Data Availability

The datasets analyzed in the current study are available from the corresponding author on reasonable request.
